# Utility of Serum miR-125b as a Diagnostic and Prognostic Indicator and Its Alliance with a Panel of Tumor Suppressor Genes in Epithelial Ovarian Cancer

**DOI:** 10.1371/journal.pone.0153902

**Published:** 2016-04-19

**Authors:** Mariyam Zuberi, Imran Khan, Rashid Mir, Gauri Gandhi, Prakash Chandra Ray, Alpana Saxena

**Affiliations:** 1 Department of Biochemistry, Maulana Azad Medical College and Associated Hospitals, New Delhi, India; 2 Department of Surgery, University of Illinois at Chicago, Chicago, Illinois, United States of America; 3 Prince Fahd Bin Sultan Research Chair, Faculty of Applied Medical Sciences, University of Tabuk, Saudi Arabia, Tabuk-71491; 4 Department of Gynaecology and Obstetrics, Lok Nayak Hospital, New Delhi, India; SAINT LOUIS UNIVERSITY, UNITED STATES

## Abstract

MicroRNAs (miRNAs) have been found to be dysregulated in epithelial ovarian cancer (EOC) and may function as either tumor suppressor genes (TSGs) or as oncogenes. Hypermethylation of miRNA silences the tumour suppressive function of a miRNA or hypermethylation of a TSG regulating that miRNA (or vice versa) leads to its loss of function. The present study aims to evaluate the impact of aberrant microRNA-125b (miR-125b) expression on various clinicopathological features in epithelial ovarian cancer and its association with anomalous methylation of several TSGs. We enrolled 70 newly diagnosed cases of epithelial ovarian cancer, recorded their clinical history and 70 healthy female volunteers. Serum miR-125b levels were determined by quantitative reverse transcription polymerase chain reaction (qRT-PCR) and the methylation status of various TSGs was investigated by methylation specific PCR. ROC curves were constructed to estimate the diagnostic and prognostic usefulness of miR-125b. The Kaplan—Meier method was applied to compare survival curves. Expression of miR-125b was found to be significantly upregulated (p<0.0001) in comparison with healthy controls. The expression level of miR-125b was found to be significantly associated with FIGO stage, lymph node and distant metastasis. ROC curve for diagnostic potential yielded significant AUC with an equitable sensitivity and specificity. ROC curves for prognosis yielded significant AUCs for histological grade, distal metastasis, lymph node status and survival. The expression of miR-125b also correlated significantly with the hypermethylation of TSGs. Our results indicate that DNA hypermethylation may be involved in the inactivation of miR-125b and miR-125b may function as a potential independent biomarker for clinical outcome in EOC.

## Introduction

Ovarian cancer is the leading cause of cancer related deaths worldwide. The high rate of mortality may be due to difficulties in diagnosing it at an early stage and lack of effective treatments for patients with an advanced or recurrent disease[1]. Therefore, there is a critical prerequisite for developing prognostic and predictive markers to detect it early and to help optimize treatment.

MicroRNAs (miRNAs) are a class of small non-coding RNA molecules that could induce post-transcriptional silencing of target genes by interacting with their 3′-UTR (untranslated region), thus regulating many important biological processes such as cell development, differentiation and apoptosis[2]. Taking into account the important regulatory roles miRNA play in cancer development and progression, several studies have been conducted to identify miRNA as potential biomarkers for cancer diagnosis, prognosis and personalized therapy[3].

Aberrant miRNA expression has been frequently observed in various types of human tumors. Studies suggest that miRNAs may function as either tumor-suppressor genes (TSGs) or oncogenes[4]. Recent studies reported dysregulation of miR-125b in various cancers[5,6]. However, the mechanism by which miR-125b contributes to epithelial ovarian cancer (EOC) has not been documented and is still relatively unclear. High expression of miR-125 had been observed in gliomas and prostate cancer[7,8] while miR-125 levels were seen to decrease in breast and gastric cancer[9,10]. To date, the expression level of miR-125b in epithelial ovarian cancer is not clear yet. As per the putative promoter region, the promoter of miR-125b is embedded in CpG island[11].

Considering the above findings, we tested the expression of miR-125b in serum of epithelial ovarian cancer patients to elucidate its utility as a diagnostic/prognostic marker. We also analysed the methylation pattern in a panel of tumour suppressor genes such as DAPK1, p16, RASSF1A, PTEN, BRCA1 and p14 and explored its association with the aberrant expression of miR-125b.

## Materials and Methods

### Patient Samples

Blood samples were procured from clinically diagnosed epithelial ovarian cancer patients. The patients (n = 70) were recruited from the Department of Gynaecology and Obstetrics, Lok Nayak Hospital, New Delhi between June 2012 and October 2014. Equal number (n = 70) of female volunteers (age matched) served as controls. Serum was separated by centrifugation. All samples were collected and stored at -70°C and thawed immediately before assay. Complete clinical data and current therapy plan was obtained. The purpose of the sampling was explained to all patients and written informed consent was obtained prior to enrolment. The study was approved by the Institutional Ethics Committee at Maulana Azad Medical College, New Delhi, India.

### DNA Extraction

Genomic DNA was isolated from blood specimens of patients and controls by using the Geneaid DNA Isolation Kit (Taiwan) in accordance with the manufacturer's instructions. The quality and integrity of DNA from these tissues were checked by electrophoresis on 1% agarose gel, quantified spectrophotometrically, and then stored at −20°C for further use.

### Bisulfite Modification of DNA

Bisulfite treatment was carried out using the BisulFlash DNA Modification Kit (Epigentek, USA) according to the manufacturer’s guidelines. This procedure modifies unmethylated cytosines to uracil nucleotides but does not modify methylated cytosine nucleotides. Bisulphite conversion of DNA was carried out at 95°C for 20 minutes during which unmethylated cytosines were converted into uracil completely. This was followed by the converted DNA clean-up and storage at -80°C prior to use.

### Methylation-specific PCR

Methylation-specific PCR (MSP) is sensitive and specific for methylation of virtually any block of CpG sites within a CpG island. The frequency of CpG sites in CpG islands renders this technique useful and extremely sensitive for such regions. Bisulfite treated DNA was amplified with specific PCR primers (S1 Table) that distinguish methylated and unmethylated DNA. These primers amplify different sized products for DAPK1, p16, RASSF1A, PTEN, BRCA1 and p14 (for methylated and unmethylated sequences) as shown in the supplementary file (S1 Fig). PCR was carried out in a 25 μL mixture containing 10 μL of Dream Taq Master Mix (Fermentas) containing optimized buffer, 4 mM MgCl_2_, recombinant Taq DNA polymerase, 0.4 mM of each dNTP, 0.6 μM of each primer, and 4.0 μL of bisulfite-treated template DNA.

### RNA Extraction, polyadenylation and cDNA synthesis

RNA from serum samples was isolated by Trizol method and stored in RNase free tubes at -70°C. The quality and integrity of the RNA was determined by the A_260/280_ ratio. The Poly-A tailing was carried out using Poly A Polymerase enzyme and rATP (Agilent, Cat#600036). Reverse Transcriptase and other necessary reagents for cDNA synthesis were subsequently added to convert the poly(A) tailed miRNAs into cDNA using an oligo-dT adapter primer provided with the kit. The adapter primer has a unique sequence at its 5’ end which allows amplification of cDNAs in real-time qRT-PCR reactions.

### miRNA detection by qPCR

Relative quantitative real-time PCR (qPCR) was used to measure the expression of miRNA. qPCR was carried out on a Rotor Gene (Qiagen), using Maxima SYBR Green qPCR mastermix (Fermentas), miR-125b specific forward primer (Qiagen) (Table 1), a common universal reverse primer and snU6 as an endogenous control. 100 ng of the cDNA product amplified in the reverse transcription step above were quantified in a 20 μl reaction volume using the following amplification program: DNA *Taq* polymerase activation at 95°C for 5 min, followed by 40 amplification cycles of denaturation at 95°C for 10 s, annealing at 61°C for 20 s, and elongation at 72°C for 10 s. Lastly, a melting curve was generated by taking fluorescent measurements every 0.5°C for 25 s from 50°C until 95°C to ensure a single PCR product. The real-time PCR experiments were repeated atleast thrice.

**Table 1:** Sequence of mature miR-125b primer used.

Name	Chromosomal location	Pre-miRNA length	Sequence (mature miRNA)
miR-125b	Chromosome 11	88	**5'UCCCUGAGACCCUAACUUGUGA**

The Ct values of housekeeping U6 snRNA and test mir-125b were used to calculate the delta Ct (ΔCt) values between test and reference genes in both normal healthy controls and test patients. Delta delta Ct (ΔΔCt) values between healthy controls and patients were based on difference in ΔCt values between the two sets. This was used to calculate the exponential difference based on 2^–ΔΔCt^. The values were normalized and expressed in terms of fold expression relative to healthy controls.

### Statistical Analysis

The correlations between the methylation levels of tumour suppressor genes and the clinical and pathological parameters were analyzed with Chi-square test or Fisher exact probability analysis. The difference of miR-125b expression levels between epithelial ovarian cancer patients and controls was examined by Student's *t*-test. ROC curves were constructed using XLSTAT software. Survival analysis was performed by using Kaplan—Meier plots and log—rank (Mantel-Cox) test. Data analysis for qPCR was performed by the comparative threshold cycle (Ct) method. Each sample was examined in triplicate and the amounts of the PCR products produced were normalized to the internal controls. Gene and miRNA expression levels were obtained by relative quantification using the 2^(-ΔΔCt)^ method. Findings greater or lesser than 1 were considered to indicate overexpression or underexpression, respectively. All values were standardized relative to the normal control values, which were represented as a value of 1. The relationship of miR-125b and methylation status of TSGs was examined by Mann-Whitney *U* test. SPSS version 16.0 for Windows (SPSS Inc, IL, USA) and GraphPad Prism 6 for Windows (Version 6.05) were used for statistical analysis. Results were regarded as significant when *p* was <0.05.

## Results

### Patient characteristics

Clinical-pathological features including age, histological grade and type, tumor size, menopausal status, FIGO stage, lymph node status, CA125 levels etc. are shown in Table 2. To elucidate the expression of miR-125b on the onset of cancer, epithelial ovarian cancer patients were divided into two groups, ≤ 45 years (57.1%) and > 45 years (42.8%). Cases were divided according to the FIGO staging of EOC and histopathological types.

**Table 2:** Association between miR-125b expression and clinicopathological characteristics in epithelial ovarian cancer.

Parameter	Number n(%)	miR-125b ExpressionHigh (n)(%)	p-value
**Age (years)**			
≤45	40 (57.1)	17 (42.5)	0.13
>45	30 (42.8)	19 (63.3)	
**Histological grade**			
Well	25 (35.7)	12 (48.0)	**0.04**
Moderate	26 (37.1)	18 (69.2)	
Poor	19(27.1)	6 (31.5)	
**Histological type**			
Mucinous	36(51.4)	18 (50.0)	0.31
Serous	17 (24.2)	11 (64.7)	
Papillary	8 (11.4)	2 (25.0)	
Others	9	5 (55.5)	
**Tumor size**			
<10 cm^3^	5(7.1)	4 (80.0)	0.22
10–50 cm^3^	7(10.0)	5(71.4)	
50–200 cm^3^	14(20.0)	9 (64.2)	
200–500 cm^3^	17(24.2)	7 (41.1)	
>500 cm^3^	27(38.5)	11 (40.7)	
**FIGO stage**			
I	23(32.8)	23 (100.0)	**<0.0001**
II	10(14.2)	10 (100.0)	
III	19(27.1)	2 (10.5)	
IV	18(25.7)	1 (5.5)	
**Distant metastasis**			
Absent	52(74.2)	35 (67.3)	**0.002**
Present	18 (25.7)	1 (5.0)	
**Lymph node status**			
Absent	51(72.8)	33 (64.7)	**0.001**
Present	19(27.1)	3 (15.7)	
**Menopause**			
Pre	39(55.7)	17 (43.5)	0.21
Post	31(44.2)	19 (61.2)	
**Nulliparity**			
Absent	51(44.2)	30 (58.8)	0.07
Present	19(27.1)	6 (31.5)	
**Serum CA125 (U/ml)**			
≤35	9(11.4)	5 (55.5)	0.92
>35	61(88.5)	31 (50.8)	
**Hemoglobin (gm/dL)**			
≤12	59(84.2)	30 (50.8)	0.92
>12	11(15.7)	6 (54.5)	

### Distribution of miR-125b expression pattern in cases and controls

Table 3 depicts the allocation of miR fold change expression amongst cases and controls. There was significant difference **(p<0.0001)** observed with serum miR-125b expression in epithelial ovarian cancer. One-sample t-test was used to evaluate the miRNA expression between patients and healthy controls. The level of miR expression in controls was set to one and then calculated miR expression in matched ovarian carcinoma samples. The serum expression of miR-125b was found to be 5 folds less in the controls than the corresponding epithelial ovarian cancer patient (Table 3).

**Table 3:** Distribution of fold change expression pattern of miR-125b in EOC patients.

miRNA	Number of samples (N)	Mean fold change expression ± SD	95% C.I	p-value
miR-125b	70	5.25±1.04	0.845–1.66	<0.0001

### Expression of miR-125b and its diagnostic potential in epithelial ovarian cancer

**Fig 1:**
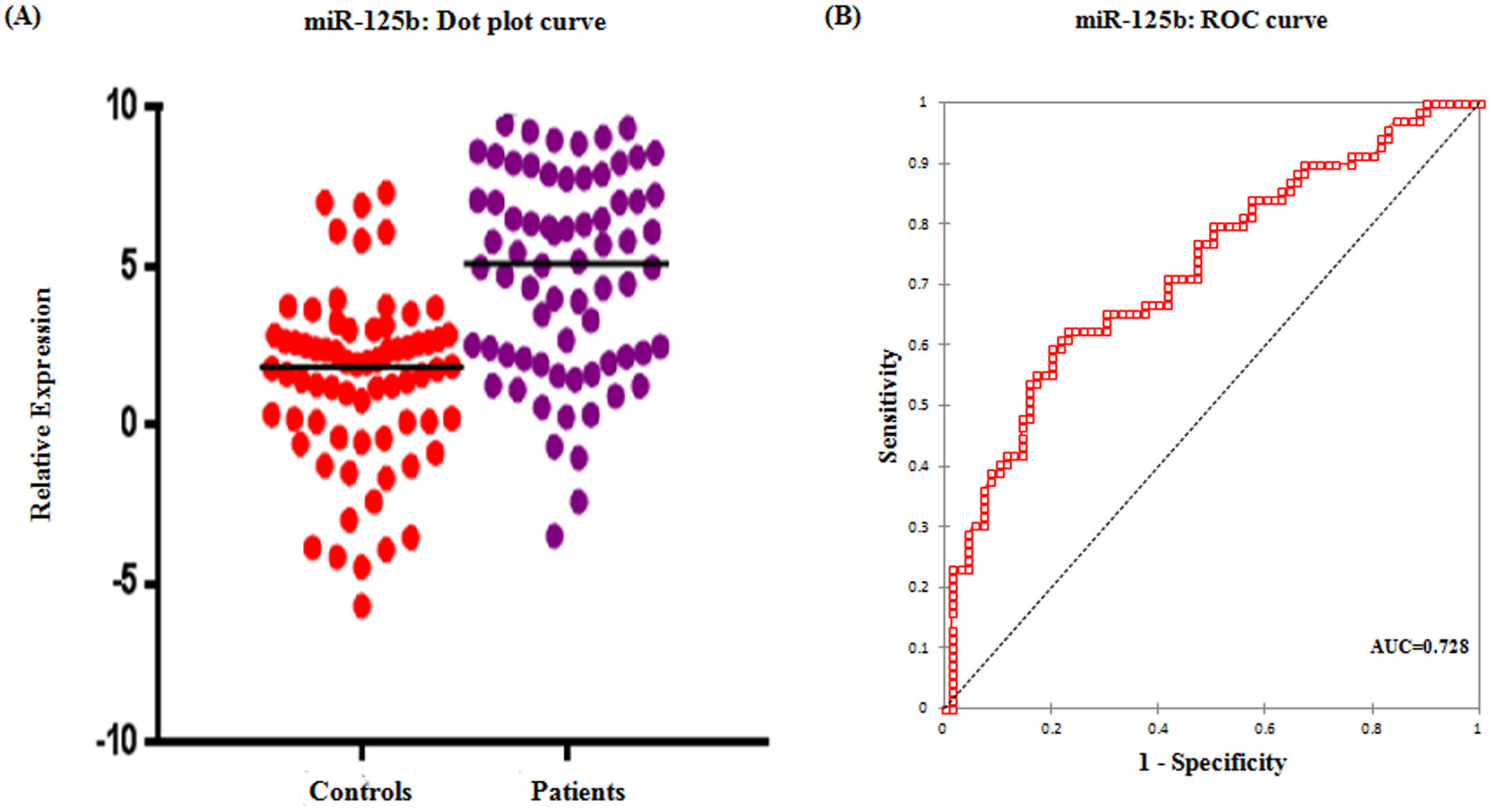
Serum expression of miR-125b. (A) Dot plot showing the relative expression of miR-125b in patients and controls (B) ROC curve for miR-125b exhibiting its diagnostic potential in epithelial ovarian cancer.

Real time relative quantification analysis showed more than 5 fold increase in serum miR-125b expression among epithelial ovarian cancer patients than healthy controls (Table 3). ROC curve analyses were performed to evaluate the predictive power of miR-125b for malignant ovarian cancer and illustrated in Fig 1. Relative expression of the serum miR-125b could discriminate patients with malignant ovarian cancer from healthy controls, with a power AUC of 0.728 (95% CI = 0.64–0.81) at a sensitivity of 62.3% and specificity of 77.1% from a cut-off score of 3.76.

### miR-125b upregulation in relation to clinicopathological features

**Fig 2:**
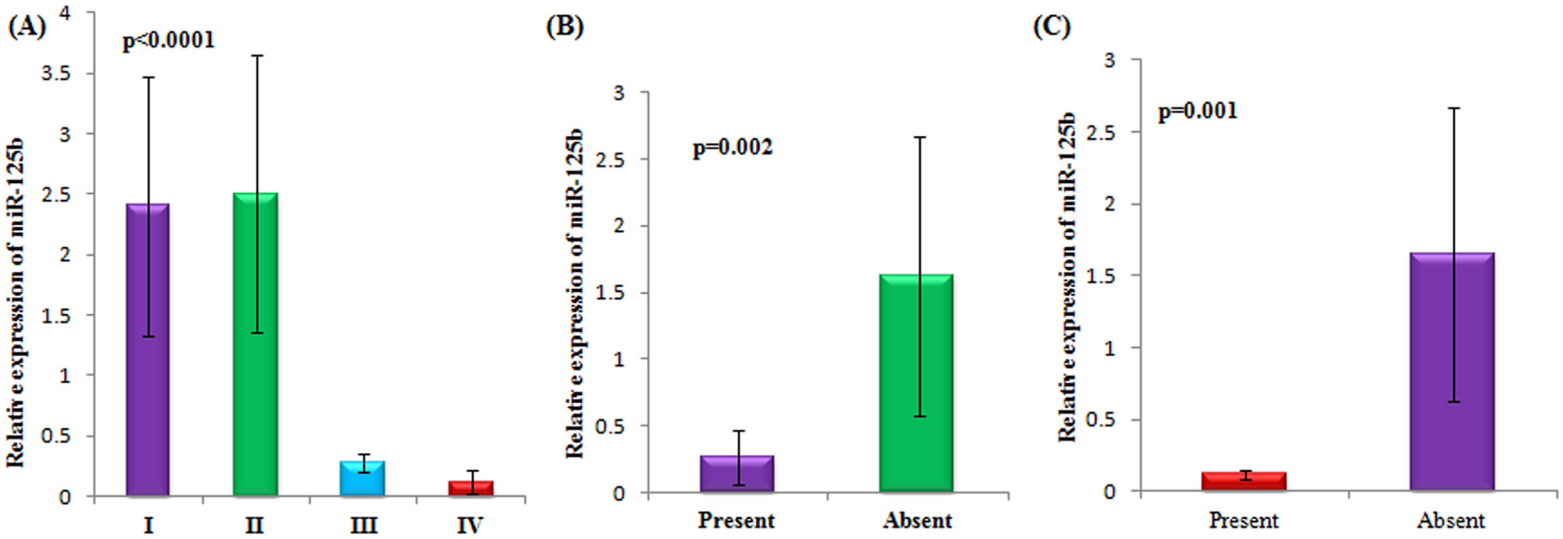
Association of serum miR-125b and clinicopathological characteristics in epithelial ovarian cancer patients with respect to A)Stage, (B)Lymph node metastasis and (C)Distant metastasis status.

Table 2 depicts the correlation between miR-125b expression and clinicopathological characteristics of epithelial ovarian cancer patients. Around 64% of patients of >45 years of age showed high expression of miR-125b. Almost 70% patients having moderate grade tumour exhibited increased expression of miR-125b apart from 48% patients with well differentiated tumour and 32% poorly differentiated tumour. Elevated expression of miR-125b was seen in around 65% serous adenocarcinoma patients and about 50% patients with mucinous adenocarcinoma. When correlated with tumour size, high expression of miR-125b was observed when tumour was at its initial stage i.e. <10cm^3^. Moreover, all patients in the early stage (stage I and II) showed high expression of miR-125b. Patients having no metastasis ie, involving those patients in the early stage, summed up almost 67% of patients with increased expression of miR-125b as compared to patients (5%) with metastatic disease. Similarly, 65% patients with no lymph node metastasis showed higher expression of miR-125b. Menopausal status, nulliparity, serum CA125 levels and haemoglobin (Hb) concentration did not associate significantly with the expression of miR-125b. Fig 2 demonstrates the significant relationship with respect to stage, lymph node metastasis and distant metastasis status with the expression of miR-125b in epithelial ovarian cancer patients.

#### miR-125b with respect to stage of epithelial ovarian cancer

A lucid demarcation was observed with respect to the expression of miR-125b and the FIGO stages in epithelial ovarian cancer. All patients in Stage I and stage II presented higher expression of miR-125b while patients in stage III and IV counted merely 10% and 5% of patients with increased expression respectively (Fig 2A).

#### miR-125b is upregulated in non metastasized tumours

A statistically significant **(p = 0.002)** correlation was observed between the metastasis status of epithelial ovarian cancer patients and miR-125b expression. Expression of miR-125b was found to be specifically increased in patients with no metastasis (Fig 2B).

#### miR-125b is upregulated in patients with no lymph node metastasis

As with distant metastasis, patients with no lymph node metastasis showed a statistically significant **(p = 0.001)** correlation with the expression of miR-125b (Fig 2C).

### Expression of miR-125b and its prognostic potential in epithelial ovarian cancer

miR-125b was analyzed for its disease progression, prognostic significance and survival outcome in EOC. miR-125b was evaluated for disease progression by the association of its expression with the FIGO stages and various other clinicopathological parameters of epithelial ovarian cancer. The prognostic significance was elucidated by ROC curves. To use ROC curve analysis, the clinical and tumor characteristics were made binary and therefore dichotomized. Stage was dichotomized as early (I+II) or late (III+IV), tumor grade as low (G1+G2) or high (G3), node involvement as no lymph node involvement or any lymph node involvement, distant metastasis as present or absent, survival as death due to epithelial ovarian cancer or other (censored/alive).

**Fig 3:**
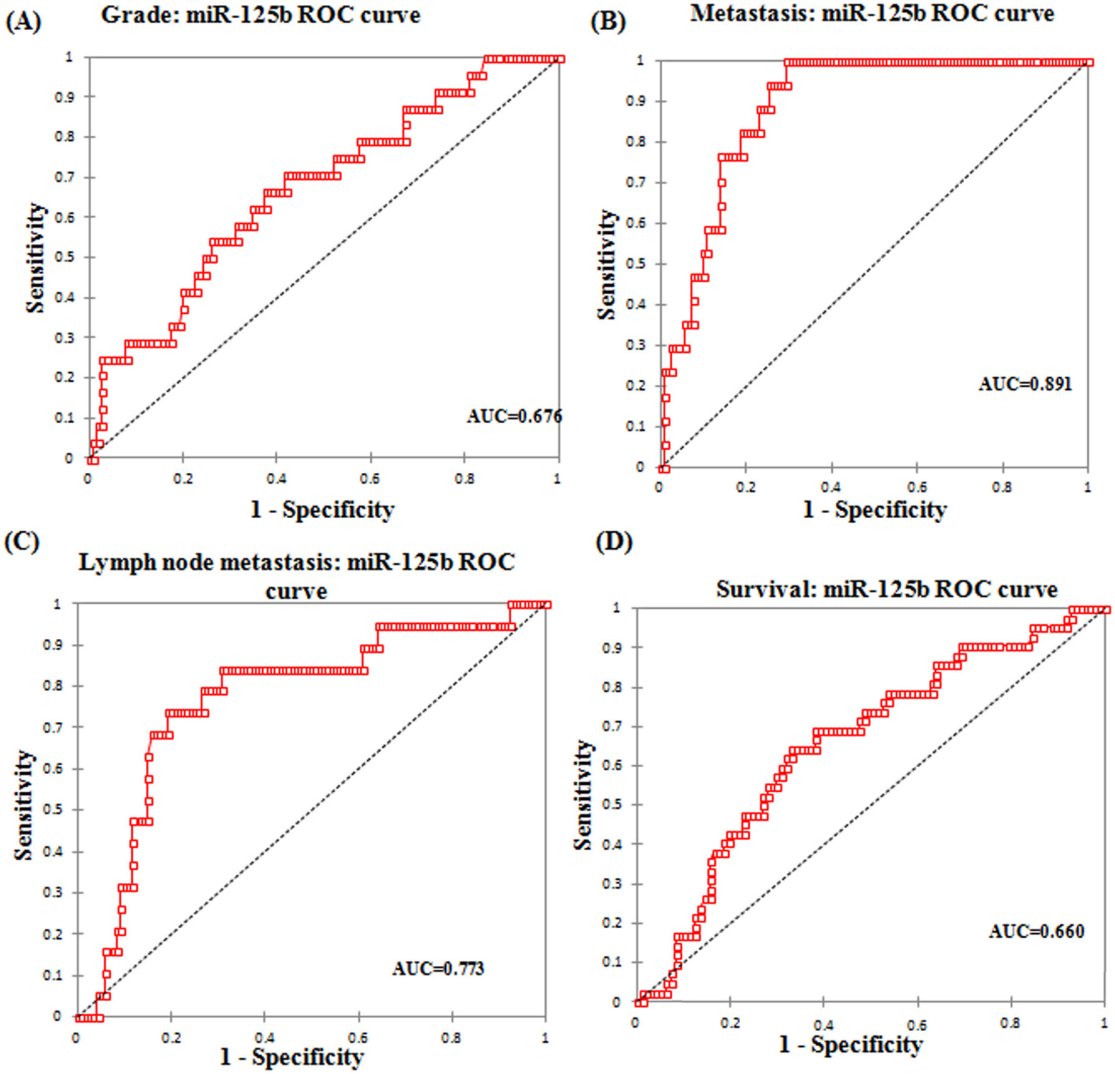
Area under curve (AUC) of receiver operating characteristic (ROC) for miR-125b corresponding to (A)Tumor grade (B)Metastasis (C)Lymph node status and (D)Survival of EOC patients.

The expression of miR-125b and histological grade yielded a significant AUC of 0.676 (95% CI: 0.558–0.793) with a sensitivity of 66.7% and specificity of 62.6% from an optimal cut-off value of 3.91 (Fig 3A) (Table 4). ROC curve for distant metastasis yielded an AUC of 0.891 (95% CI: 0.838–0.944) with a sensitivity and specificity of 94.1% and 70.5% respectively at an optimal cut-off value of 4.34 (Fig 3B). An AUC of 0.773 (95% CI: 0.658–0.887) was obtained in ROC curve with respect to lymph node metastasis and at an optimal cut-off value of 5.85, the sensitivity was 73.7% and specificity was 80.8% (Fig 3C). ROC curve for survival yielded an AUC of 0.660 (95% CI: 0.564–0.756) with a sensitivity of 64.3% and specificity of 67.0% at an optimal cut-off score of 3.91 (Fig 3D).

**Table 4:** AUC for ROC curve corresponding to the diagnostic value of miR-125b in EOC.

Parameter	AUC	Standard Error	95% C.I.
Histological grade	0.676	0.060	558–0.793
Distant metastasis	0.891	0.027	0.838–0.944
Lymph node	0.773	0.059	0.658–0.887
Survival	0.660	0.049	0.564–0.756

**Fig 4:**
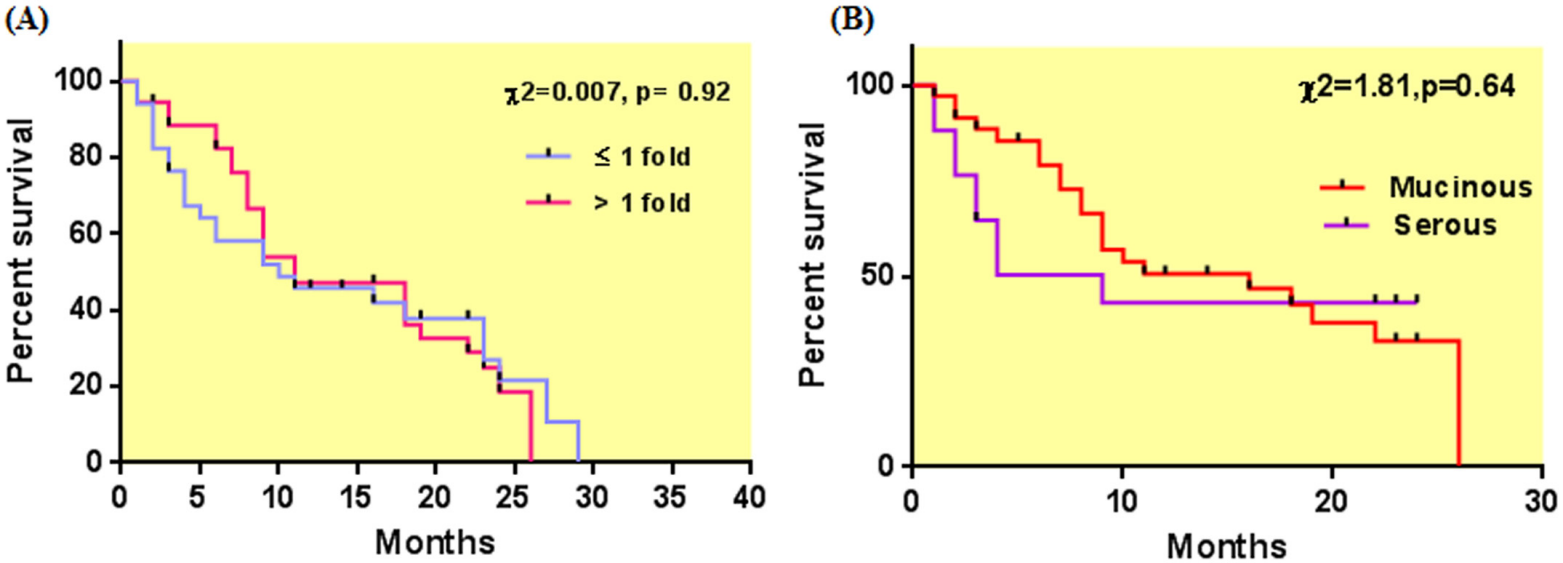
Kaplan Meier survival curve with respect to (A) fold change (B) mucinous and serous histopathological subtypes.

The survival analysis was done by Kaplan-Meier curve and log-rank test (Fig 4A). There was no significant association found between the miR-125b expression and overall survival of patients with ≤1 fold expression of miR-125b. However, when histological subgroups were estimated for survival separately, it was found that patients with mucinous histotypes had a survival time of 16 months whereas, patients with serous histotypes exhibited a survival time of 9 months (Fig 4B).

### Expression of miR-125b in association with promoter hypermethylation of tumour suppressor genes

**Fig 5:**
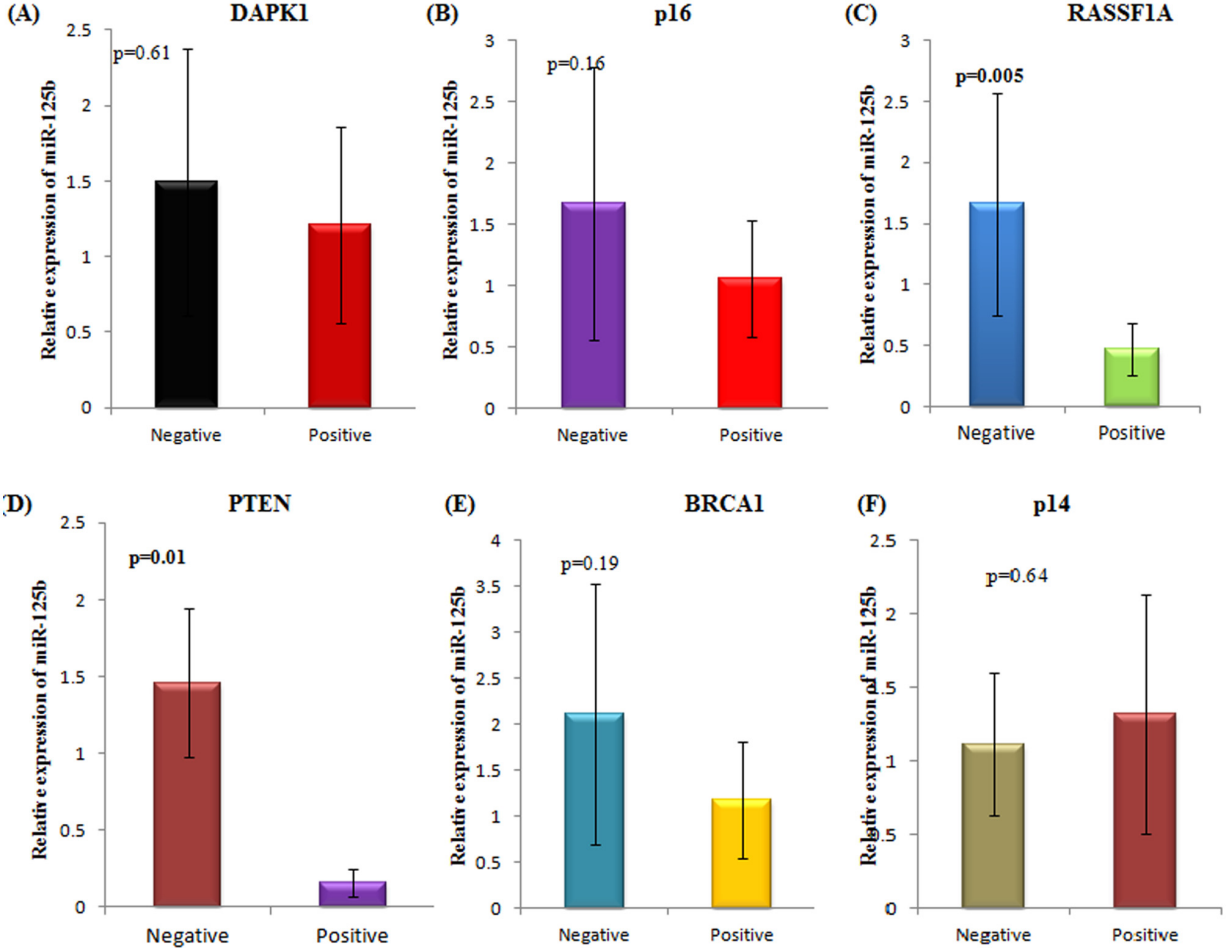
Association of miR-125b expression with the promoter hypermethylation of a panel of tumour suppressor genes. (A)DAPK1 (B) p16 (C) RASSF1A (D) PTEN (E) BRCA1 (F) p14. Positive and negative depicts the presence or absence of promoter hypermethylation of the specified tumour suppressor gene respectively.

The relative expression of miR-125b was assessed in relation to the promoter hypermethylation of well known tumour suppressor genes—DAPK1, p16, RASSF1A, PTEN, BRCA1 and p14. Out of these, RASSF1A and PTEN genes showed a statistically significant correlation between the aberrant hypermethylation of their promoter and the relative expression of miR-125b in epithelial ovarian cancer patients. Patients with RASSF1A promoter hypermethylation demonstrated downregulation of miR-125b expression **(p = 0.005)** (Fig 5). Similarly, patients with PTEN promoter hypermethylation showed downregulation of miR-125b expression **(p = 0.01)**. No other tumour suppressor gene exhibited a statistically significant association with miR-125b expression.

## Discussion

The development of epithelial ovarian carcinogenesis is a multistep procedure entailing the dysregulation of oncogenes and tumor suppressor genes. Contemporary studies have reported that microRNA-associated transcriptionally regulated gene expression plays a crucial role in the initiation, development and progression of epithelial ovarian cancer, by keeping a regulatory check on the cell proliferation, cell cycle, apoptosis, and metastasis. miR-125b has been reported to be involved in multiple cancers[12]. miR-125b functions either as an oncogene or tumor suppressor gene. It is upregulated and displays oncogenic potential in some cancers as it triggers cell division, while hampering apoptosis. On the other hand, it is overtly downregulated in various other cancers. Our understanding with respect to the contradictory roles of miR-125b in plethora of cancers is still limited. One aspect of our aim was to explore the aberrant expression of miR-125b in epithelial ovarian cancer in relation to the clinicopathological attributes. The other aspect was to test the expression of miR-125b with reference to the anomalously methylated TSGs in order to vividly conclude their possible role in either regulating or being regulated by miR-125b.

miR-125b is thought to target the hallmarks of cancer in one way or the other, regulating multiple genes involved in the pathway, leading to the development of the cancer phenotype. Aberrant DNA methylation has a critical outcome on epithelial ovarian cancer initiation and progression. Several investigators have looked for the hypermethylation of the miRNA promoter in different cancers as a reason for the inactivation of that particular miRNA and highlighting the putative role of epigenetic disruption in producing the aberrant patterns of expression of miRNAs in cancer cells[13]. Several lines of evidence support the idea that dysregulation of miRNA can lead to aberrant DNA methylation in cancer and that miRNAs able to regulate *DNMT* genes are reportedly downregulated in cancer[14,15]. miRNAs which act as tumour suppressors have been found to be methylated in tumour cells indicating the significance the studying the two forces together- epigenetics and miRNAs[16]. Also, the DNA methylation profile of tumors can be used as a signature to define tumor type, clinical prognosis and treatment response[17,18]. miRNAs transcribed from CpG islands undergo DNA methylation-associated repression with a similar chromatin context to coding genes, such as binding of the transcriptional repressor methyl-CpG-binding domain proteins[19,20]. Such genetic unmasking studies have paved way in identifying the mechanisms responsible for causing the cancer havoc at the molecular level. Based on similar lines, we selected a panel of tumour suppressor genes, which are known to be susceptible in epithelial ovarian cancer to investigate whether their aberrant methylation directly or indirectly affects the expression of miR-125b which in turn leads to the development of cancer. Of the studied tumour suppressor genes, RASSF1A and PTEN genes emerged as likely candidates which might be playing a crucial role in modulating the expression of miR-125b. Poliseno et al. have demonstrated the role of PTEN transcripts in modulating miRNA targeting by examining PTENP1 levels[21]. The identification and validation of numerous *PTEN*-targeting microRNAs indicates that post-transcriptional regulation plays a central role in determining PTEN abundance in cancer cells[22–25]. Cells are highly sensitive to even slight decrease in PTEN levels, highlighting the importance of microRNA-mediated PTEN regulation in cancer[26]. RASSF1A is a tumor suppressor that is a negative regulator in the RAS-MAPK pathway and has reduced expression in various cancers. It has been proposed that RASSF1A participates in cell proliferation and apoptosis by regulating the MAPK pathway and has effects on carcinogenesis[27]. Another study by Banno et al. reported that reduced expression of RASSF1A gene occurs by hypermethylation or suppression of gene expression by miRNAs. Expression of the miRNA itself may be increased or decreased by promoter methylation, based on differences between normal and cancerous cells[28]. Hence our study has extrapolated these results and might give a new direction to examine the expression of miR-125b in a new dimension.

Recent technological advancements have aided the detection of miRNAs at optimum sensitivity and specificity. Circulating miRNAs are emerging as promising diagnostic biomarkers for cancer though their usefulness for detecting early neoplasms still remains unclear. miR-125b has been shown to target multiple genes which are more or less relevant in the formation of tumour in different cell types. miR-125b acts as classical oncogene in a variety of tumours where its overexpression may lead to the suppression of a tumour suppressor gene or block its apoptotic machinery. This is further supported by the finding that miR-125b expression levels are correlated with reduced survival rates[29,30]. A recent study by Yamada et al. has also reported an elevated level of miR-125b in colorectal cancer. They identified miR-125b as an early detection biomarker in colorectal cancer owing to the considerable AUC (0.806) in the ROC curve[31]. Giray et al. found an upregulation of miR-125b expression in hepatocellular carcinoma and suggested that it could serve as a novel, non invasive biomarker in this cancer[32]. Jiang and colleagues also showed an upregulation of miR-125b in vivo and in vitro in endometrial carcinoma[33]. In addition, overexpression of miR-125b has been investigated in several cancers such as pancreatic cancer[34], prostate cancer[35], breast cancer[36,37], lung cancer[38], glioblastoma[39], oligodendroglial cancer[7] amongst other cancers. Conversely, miR-125b has been shown to be downregulated in a number of tumours such as bladder cancer[40], esophageal cancer[41], osteosarcoma[42], prostate cancer[43], liver cancer[44], breast cancer[45], non-small cell lung cancer (NSCLC)[46], breast cancer[47], gastric cancer[48], glioblastoma[49] etc. In ovarian cancer, contradictory studies on miR-125b have made it a necessity to survey its role profoundly. Lee et al. reported a downregulation of miR-125b in ovarian cancer tissue and cell lines compared to healthy controls[50]. Luo and colleagues have also recently reported a downregulation of miR-125b in ovarian cancer tissue and cell lines[51]. Gadducci et al. also observed similar results[52]. He et al. suggested a novel therapeutic approach using miR-125b mimic in the treatment of ovarian cancer[53]. Guan et al. reported that overexpression of miR-125b resulted in the inhibition of ovarian cancer cell proliferation[54]. On the other hand, Kong and colleagues reported an upregulation of miR-125 in ovarian cancer and suggested that up-regulation of miR-125b expression contributes to cisplatin resistance through suppression of Bak1 expression[55]. Diverse expression of miR-125b was reported in drug-resistant ovarian cancer cell lines by Sorrentino et al[56].

The association of miR-125b with various clinicopathological features reflect that miR-125b may function as an early biomarker for ovarian cancer. The elevated expression in the early stages as compared to the advanced stage speaks volumes of its significance in indicating the onset of the disease. The expression of miR-125b in relation to the distant metastasis status and lymph node status suggest its possible potential in the early diagnosis of ovarian cancer.

## Conclusion

Increasing number of studies have shown that miR-125b plays a crucial role in diverse cellular processes and many diseases especially carcinomas. It is noteworthy that the functions of miR-125b are controversial in different types of cancers. Hence, the controversial properties of miR-125b in various tumors suggest that miR-125b have distinct functions in cancer pathogenesis and progression, while the underlying mechanisms on different cell context need further validation. The advances that miR-125b holds in clinical applications need a closer look for future treatment of cancer.

## Supporting Information

S1 Fig Electrophoretic band pattern for hypermethylation for a panel of tumor suppressor genes visualized on 2% agarose gel under UV transillumination. (EPS)

S1 Table Sequence of primers with annealing temperatures and band size used for MS-PCR. (RTF)
