# Supplementary material for: Utility of Serum miR-125b as a Diagnostic and Prognostic Indicator and Its Alliance with a Panel of Tumor Suppressor Genes in Epithelial Ovarian Cancer
Source: PLoS One. 2016 Apr 19;11(4):e0153902. doi: 10.1371/journal.pone.0153902 (PMC4836713; doi:10.1371/journal.pone.0153902)
Supplement: S1 Table — (RTF) [file pone.0153902.s002.rtf]

Gene	Forward primer	Reverse primer	AT
 (°C)	Size (bp)	
DAPK1(M)	GGATAGTCGGATCGAGTTAACGTC	CCCTCCCAAACGCCGA	62	103	
DAPK1(U)	GGAGGATAGTTGGATTGAGTTAATGTT	CAAATCCCTCCCAAACACCAA	62	98	
p16 (M)	TTATTAGAGGGTGGGGCGGATCGC	GACCCCGAACCGCGACCGTAA	65	150	
p16 (U)	TTATTAGAGGGTGGGGTGGATTGT	CAACCCCAAACCACAACCATAA	60	151	
RASSF1A(M)	GCTAACAAACGCGAACCG	CCCTCCCAAACGCCGA
	60	169	
RASSF1A(U)	GGAGGATAGTTGGATTGAGTTAATGTT	GGTTTTTGTGAGTGTGTTTAG	60	169	
PTEN (M)	GGTTTCGGAGGTCGTCGGC	CAACCGAATATTAACTACTACGACG	61	155	
PTEN (U)	TGGGTTTTGGAGGTTGTTGGT	ACTTAACTCTAAACCACAACC	61	155	
BRCA1 (M)	GGTTAATTTAGAGTTTCGAGAGACG	TCAACGAACTCACGCCGCGCAATCG
	59	182	
BRCA1 (U)	GGTTAATTTAGAGTTTTGAGAGATG	TCAACAAACTCACACCACACAATCA	54	182	
p14 (M)	GTGTTAAAGGGCGGCGTAGC	AAAACCCTCACTCGCGACGA	58	122	
p14 (U)	TTTTTGGTGTTAAAGGGTGGTGTAGT	CACAAAAACCCTCACTCACAACAA	60	132	
S1 Table: Primer sequence sets used in MS-PCR 
